# Sharing datasets of the COVID-19 epidemic in the Czech Republic

**DOI:** 10.1371/journal.pone.0267397

**Published:** 2022-04-21

**Authors:** Martin Komenda, Jiří Jarkovský, Daniel Klimeš, Petr Panoška, Ondřej Šanca, Jakub Gregor, Jan Mužík, Matěj Karolyi, Ondřej Májek, Milan Blaha, Barbora Macková, Jarmila Rážová, Věra Adámková, Vladimír Černý, Jan Blatný, Ladislav Dušek

**Affiliations:** 1 Institute of Health Information and Statistics of the Czech Republic, Prague, Czech Republic; 2 Institute of Biostatistics and Analyses, Faculty of Medicine, Masaryk University, Brno, Czech Republic; 3 Medical Simulation Centre, Faculty of Medicine, Masaryk University, Brno, Czech Republic; 4 Faculty of Informatics, Masaryk University, Brno, Czech Republic; 5 National Institute of Public Health, Prague, Czech Republic; 6 Ministry of Health of the Czech Republic, Prague, Czech Republic; 7 Department of Preventive Cardiology, Institute for Clinical and Experimental Medicine, Prague, Czech Republic; 8 Department of Nephrology, First Faculty of Medicine, Charles University, Prague, Czech Republic; 9 Faculty of Biomedical Engineering, Czech Technical University, Kladno, Czech Republic; 10 Department of Anaesthesiology, Perioperative Medicine and Intensive Care, Masaryk Hospital in Ústí nad Labem, Ústí nad Labem, Czech Republic; 11 Jan Evangelista Purkyně University, Ústí nad Labem, Czech Republic; Universidad Internacional de La Rioja, SPAIN

## Abstract

At the time of the COVID-19 pandemic, providing access to data (properly optimised regarding personal data protection) plays a crucial role in providing the general public and media with up-to-date information. Open datasets also represent one of the means for evaluation of the pandemic on a global level. The primary aim of this paper is to describe the methodological and technical framework for publishing datasets describing characteristics related to the COVID-19 epidemic in the Czech Republic (epidemiology, hospital-based care, vaccination), including the use of these datasets in practice. Practical aspects and experience with data sharing are discussed. As a reaction to the epidemic situation, a new portal *COVID-19*: *Current Situation in the Czech Republic* (https://onemocneni-aktualne.mzcr.cz/covid-19) was developed and launched in March 2020 to provide a fully-fledged and trustworthy source of information for the public and media. The portal also contains a section for the publication of (i) public open datasets available for download in CSV and JSON formats and (ii) authorised-access-only section where the authorised persons can (through an online generated token) safely visualise or download regional datasets with aggregated data at the level of the individual municipalities and regions. The data are also provided to the local open data catalogue (covering only open data on healthcare, provided by the Ministry of Health) and to the National Catalogue of Open Data (covering all open data sets, provided by various authorities/publishers, and harversting all data from local catalogues). The datasets have been published in various authentication regimes and widely used by general public, scientists, public authorities and decision-makers. The total number of API calls since its launch in March 2020 to 15 December 2020 exceeded 13 million. The datasets have been adopted as an official and guaranteed source for outputs of third parties, including public authorities, non-governmental organisations, scientists and online news portals. Datasets currently published as open data meet the 3-star open data requirements, which makes them machine-readable and facilitates their further usage without restrictions. This is essential for making the data more easily understandable and usable for data consumers. In conjunction with the strategy of the MH in the field of data opening, additional datasets meeting the already implemented standards will be also released, both on COVID-19 related and unrelated topics.

## Introduction

Open data, i.e. “data that can be freely accessed, used, modified, and shared by anyone for any purpose”, represents a systematic approach to making selected government-produced datasets available for further manual or automatic processing in a uniform and clearly defined way [1]. At the time when the COVID-19 pandemic paralysed the entire world, providing access to data (suitably prepared from the perspective of personal data protection) plays a crucial role in providing the general public and media with up-to-date information. Open datasets represent one of the means for evaluation of the pandemic on a global level. The sources of such datasets include, for example, the WHO (World Health Organization), ECDC (European Centre for Disease Control) via Our World in Data [2], and the EU Open Data Portal [3]. Many datasets, including those published by the Johns Hopkins University [4], are available through the GitHub platform [5, 6]. Typically, websites guaranteed by governments or governmental agencies in the individual countries are used as sources of information for such international and/or global statistics. The same is true about the Czech Republic [7], presenting both visualisation and direct links to all open datasets on the COVID-19 epidemic situation on a website guaranteed by the Ministry of Health (MH). A similar approach was adopted by many other countries, including the USA [8], France [9], Germany [10], Austria [11], Spain [12], United Kingdom [13], etc. Datasets of other countries, e.g., Australia, Italy, South Africa, or Argentina, are available on GitHub [14].

From our perspective, datasets can be published as open data for several reasons: a) for the purposes of transparency (enabling verification of spending and financial management of institutions funded by the national budget, of the performance of the institutions, contracts, etc.) b) for providing complex population data (population characteristics, epidemiological statistics, medications, performance characteristics, etc.) and, last but not least, c) to offer fully aggregated data for the scientific and analytical purposes (secondarily created datasets describing groups of objects or subjects or even individual anonymised records of individuals, e.g., anonymised records of births, hospitalisations, surgeries, etc.). Some datasets are, as described below in greater detail, published in a regulated regime. Data published in this regime constitute a direct source of information about institutions, insurance payers, healthcare providers, or data intended for further processing with the aim to influence the behaviour of target subjects. In the Czech Republic, a National Catalogue of Open Data guaranteed by the MH has been developed and maintained by a joint team of the Institute of Health Information and Statistics of the Czech Republic and the Institute of Biostatistics and Analyses at the Faculty of Medicine of the Masaryk University in Brno over the last decade.

The primary aim of this paper is to describe the methodological framework including basic technical background for publishing datasets describing basic as well as advanced epidemiological characteristics related to the COVID-19 epidemic in the Czech Republic, including the use of these datasets in practice. The entire process of design, development and implementation of a robust system for tracking and reporting respiratory diseases caused by SARS-CoV-2 is based on a previously successfully used method for acquisition, processing, and presentation of information [15]. The system is based on a framework for cooperation between the Ministry of Health of the Czech Republic (MH), which collects the records from the testing points, and regional public health authorities (RPHAs). The infrastructure designed within the Czech National Health Information System (NHIS) processes these data and supports reporting of the results of testing for COVID-19 on a daily basis. Open datasets form an inherent part of the NHIS, providing information to the general public. Having this system in place has proved to be a big advantage in the current COVID-19 situation, as it was easily adaptable to suit the current requirements for information processing and presentation.

## Methods

The methodological principles leading to the publication of prepared datasets presenting basic and advanced epidemiological characteristics will be described in this section.

### The methods of data publishing and sharing

**Fig 1:**
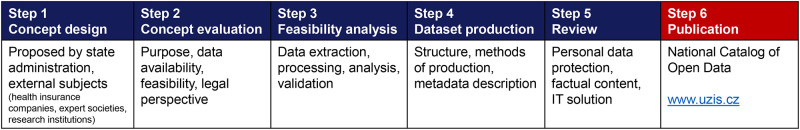
A chart of dataset production and publication.

All regimes of the data provision from NHIS strictly require a certain degree of legislative regulation and must fully meet the criteria set for NHIS by the Czech legislation, particularly Act No. 372/2011 on health services and their provision. In other words, publishing of open data cannot be misinterpreted as publication of primary records without any regulation and standardisation; the term “open data”, therefore, does not necessarily describe primary database records (the data may be aggregated, statistically processed, etc.). The dataset design, preparation, and publishing should respect the algorithm of dataset preparation shown below, which always respects several principal rules: (i) individual natural persons must not be identifiable, (ii) individual legal persons must not be identifiable unless expressly stated by the law, (iii) secondary processing must lead to the pseudonymisation of the dataset, (iv) the purpose of the dataset publication must correspond to the NHIS purpose, and (v) the standardised process of approval and publishing must be adhered to (Fig 1). The basic requirement of a comprehensive process, which is met by this approach, is to ensure the necessary completeness, validity and overall quality of data.

Publication of a dataset consists of six consequent steps that describe key phases on the preparation and implementation. Step 1: Proposal of a dataset concept in the form of short description, which can be rised by any entity; usually a state institution, a health insurance company, a professional (medical) society, a research institution, or an academic institution. Step 2: The delivered concept is reviewed from the perspective of data availability, export feasibility, design, and personal data regulations. Step 3: After approval, a methodology for the dataset processing is proposed (data export from central registries, data pre-processing and cleaning, analytical adjustments and validation mechanisms). Step 4: The dataset is generated in an open data standardised format according to the predefined scheme, including an obligatory description with metadata. Step 5: Review and validation of the dataset and its content is a mandatory procedure before publication (manual approach is always needed, as well as technical control in order to follow open data standards and best practices). Step 6: Final publication of the dataset in the National Catalogue of Open Data.

#### Freely available primary data

This publication regime is very rarely used for the publication of data from the NIHS internal systems. In this regime, the primary database records are published without any processing. In principle, this regime is only allowed for characteristics of “inanimate subjects” without any relations to personal data. Examples may include service providers as defined in the respective acts, machines, swimming pools, chemical substances or drugs, and their primary characteristics.

#### Primary data publishable after necessary processing

Such datasets are individually designed (based on proposals of groups of experts, such as clinicians or healthcare providers), reviewed, and finalised, including the descriptive parameters defining target subjects or object cohorts. Data published in this form prevent the identification of any natural or legal person. Adherence to a standardised methodology (data format unification, metadata description, publishing in central catalogue) when creating the dataset and senior expert review (advanced stakeholders in a particular healthcare domain, representatives of data management, data analysis, and development team) or approval are prerequisites for publishing in this regime. Examples of such necessary amendments include highlighting of missing values, correction of erroneous records, aggregation of records for patients’ age categories, calculation of new variables or indices from the raw data, etc. Usually, these records are completely deleted (in cases of incorrect and invalid information), directly corrected in the central database or supplemented in accordance with the methodological instructions of the register.

#### Data requiring reference interpretation—Reference statistics

Publication of statistics characterising a group (cohort) of subjects/objects (e.g. a region, period of time, patient cohort, type of medical procedures, provider category, etc.) can be also perceived as a publication of open data and/or knowledge. The user receives summary statistics and information about methods and calculation algorithms. The “reference interpretation” in this context means “a comment or summary to data output given by an expert in the field, usually given to data and values that have more sophisticated background and require careful interpretation with respect to objective uncertainties”. In the future, a special subcategory of reference departmental statistics will be formed; the list of such statistics will be approved in an executive regulation issued by the MH. These statistics must be as a rule accompanied by departmental interpretation and can be published in a way allowing the identification of an individual healthcare provider or health insurer. It is necessary to point out that such records bear a high risk of misinterpretation that could lead to erroneous decisions on the side of patients, healthcare providers, or regulatory bodies. As an example of such a parameter, we can name the hospital mortality—a figure requiring sophisticated analytical processing and interpretation with respect to case mix and other factors. Publishing such data is covered by special executive regulations.

### Technical background

From the long-term perspective, the Institute of Health Information and Statistics of the Czech Republic (IHIS CR), together with the Ministry of Health, have the aim to make as much information as possible from selected registries available as open data through the deployment and maintenance of the local Open Data Catalogue of the MH [16] and its feeding with data. These datasets are subsequently included in the National Open Data Catalogue [17], which covers and indexes (on 16 December 2020) records on datasets from other 24 local catalogues, such as the local Open Data Catalogue of the Ministry of Finance, or the Vysočina region.

The technical cornerstone for the collection and storage of data about the COVID-19 epidemic on the national level is represented by the Information System of Infectious Diseases (ISID), serving for entering individual cases at the level of regional public health authorities (RPHAs). The system supports entering of individual cases with individual diagnoses. Similar to other public health registries, ISID has been developed and operated in the unified departmental environment that successfully passed the audit by the National Security Authority and the Office for Personal Data Protection of the Czech Republic.

As a reaction to the unexpected epidemic situation, a new portal *COVID-19*: *Current Situation in the Czech Republic* was developed and launched to provide a fully-fledged and trustworthy source of information for the public and media. This online tool provides an interactive overview of the current COVID-19 spreading status in the Czech Republic [7]. This portal also contains a section devoted to datasets containing basic descriptive information on each of them. In open datasets, this information always includes metadata (the name and basic description of the dataset, date and time of last update, standardised dataset scheme); the data are available for download in CSV and JSON formats. In addition, for the purposes of supporting regional and local crisis management, the portal contains an authorised-access-only section where the authorised persons (representatives of local and regional authorities, members of emergency sevices) can—through an online generated token—safely visualise or download regional datasets with aggregated data at the level of the individual municipalities. The basic condition that the data cannot contain details facilitating direct patient identification must be of course always met.

#### The general background of open data publishing

All methods of release and processing of open data in the NHIS are stipulated by Act No. 372/2011 Coll., detailing that the data can be published or provided i) only as a summary or in an anonymized form, ii) for purposes that are not limited by legislation, and iii) for use in commercial activities, study or scientific purposes, or for public inspection of state-funded subjects. A regime for data publishing and availability is assigned by legislation to every data source, register or component of the NHIS, using categorization as follows: (i) non-public systems, (ii) systems accessible only to legislatively designated readers/editors, (iii) sources of reference statistical data for particular (named) purposes and readers, (iv) sources of statistical data published as open datasets, (v) open sources in the “open data” regime. The NHIS as a unified nationwide information system is designated mainly for: (1) Processing data on the health status of the population, on activities of healthcare providers and on their economy, on healthcare professionals and other workers in health services, for the purpose of obtaining information about the extent and quality of provided health services, for their management and for the creation of health policy. (2) Maintaining National Health Registers and processing data kept in the registers. (3) Maintaining the National Register of Health Services Providers and the National Register of Healthcare Professionals and processing data kept in the registers. (4) Performing and processing sample surveys on health status of the population, on health determinants, on the need and consumption of health services and satisfaction with the services and on expenditure on health services. (5) The needs of science and research in the field of health. (6) Processing the data listed under numbers (1) and (4) (as mentioned above) for statistical purposes and for providing data and statistical information, including information provided to international institutions, in the extent determined by this or other legal regulations.

The process of data acquisition is always in accordance with the scheme presenting dataset production and publication (see Fig 1). The crucial step consists in data standardization and metadata description of the set according to the valid rules of data set creation and best practices. Once the primary data are stored in central database, they are validated both at the level of the application layer and at the level of the database, ensuring the integrity of individual records. Subsequently, the data are anonymised (i.e., patients’ personal data are removed), and uploaded into the analytical database where they are supplemented with data from other registries (place of residence, death records, etc.). The data are subsequently transformed into a final dataset, which is exported into the CSV format and standardised. Finally, the dataset is stored at the internal cloud file server and a periodical transfer (for example once per day/week/month/year) to open data server is set up.

#### The process of COVID-19 datasets preparation, validation, and publication

The process of entering, processing, and subsequent visualisation of data describing the current epidemiological situation in relation to the new coronavirus SARS-CoV-2 requires meticulous validation. The complete workflow of how the patient suspected of being SARS-CoV-2 positive is referred by his general practitioner or regional public health authority (RPHA) for biological sampling at the testing point, how the patient record flows through the system, and how the record is further processed and stored in the Information System of Infectious Diseases (ISID), is described in detail in [15]. In terms of web-based software deployment architecture, the following tiers have been created (i) development instance, (ii) staging instance (a mirror of production instance), and (iii) production instance.

The production instance [18] of the application *COVID-19*: *An Overview of the Current Situation in the Czech Republic* for publishing freely available datasets is publicly available and provides extensive summaries shown below, in the section Results. To facilitate safe data sharing and, therefore, crisis management on both the national and regional levels (regions and municipalities with extended competence), a special interface was developed, facilitating easy access to selected authorised persons.

**Fig 2:**
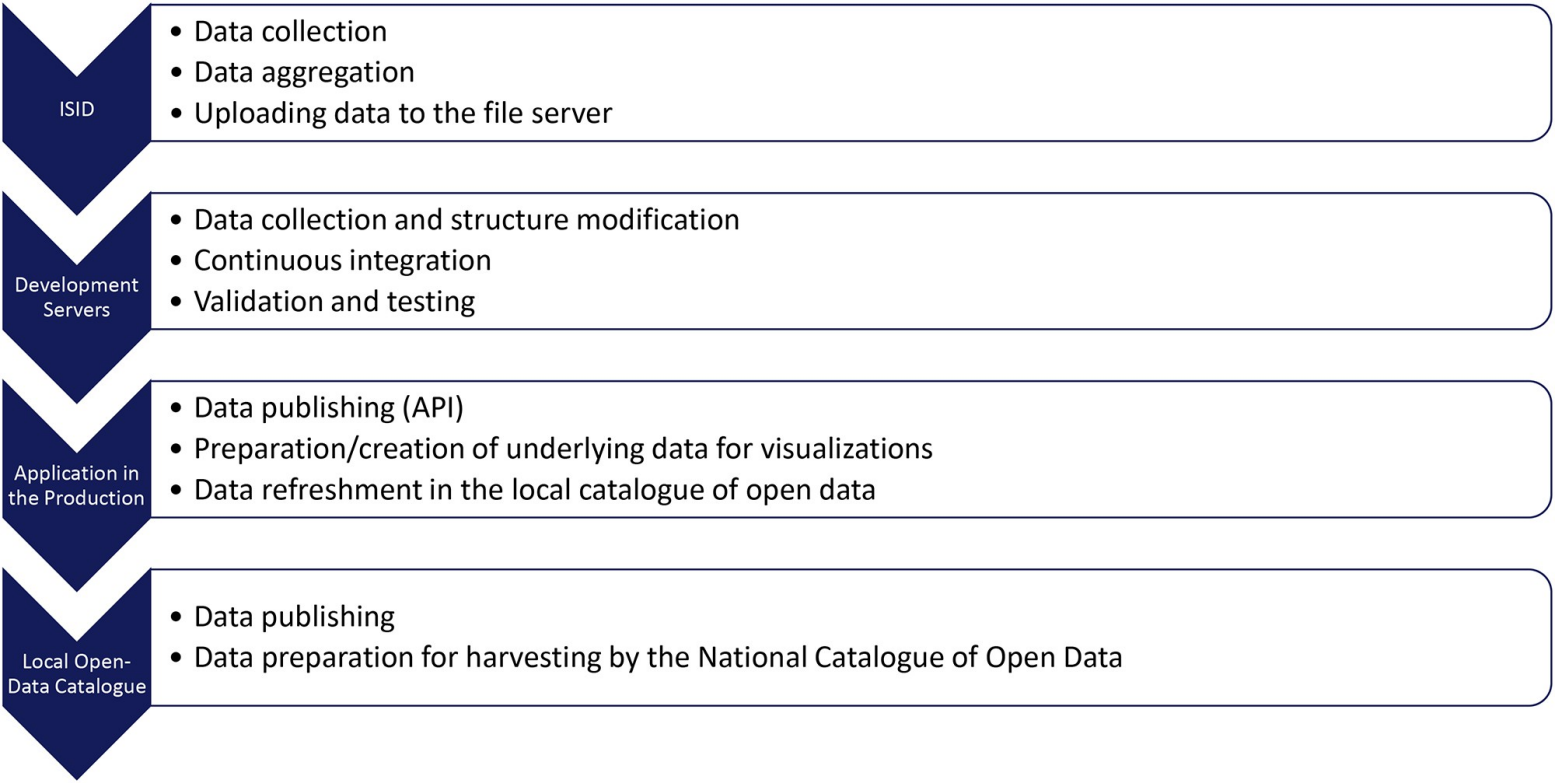
The process of data processing and publishing.

In the last stage of the dataset update (which takes place once to twice a day), the data are sent to the application interface of our local open data catalogue of the MH (covering only open data on healthcare, provided by the Ministry of Health). The main datasets are available in the CSV and JSON formats. From this catalogue, the data are harvested by the National Catalogue of Open Data (covering all open data sets, provided by various authorities/publishers, and harversting all data from local catalogues). Both catalogues contain links to datasets and their metadata (name, description, update frequency, keywords, and contact persons). The entire process is captured in Fig 2.

Some datasets might have been extended by additional attributes or their structure might have been changed after publishing (for example, data describing demography or region/district coding, which provide an additional point of view, regarding population distribution in this particular case). To maintain backward compatibility, the datasets are available in all versions using the application programming interface (API) [19]. Finally, after careful data design, processing, preparation, validation and standardisation, the data are published manually in the local catalogue using a graphical user interface, which is accesible only for authorised members of the open data team at the Ministry of Health.

## Results

### COVID-19 datasets

This section describes epidemiological characteristics that were so far published for the purposes of the online presentation and subsequent manual or automated processing on the portal *COVID-19*: *An Overview of the Current Situation in the Czech Republic*. These data are available either as open data (primary data publishable after necessary processing) or as limited access data (data requiring reference interpretation—reference statistics). The achieved results follow both methodological and well as technological perspective, which is described in the Methods section.

#### Public datasets

Depending on the dataset, the update is usually performed at 08.00 AM each day. It contains data validated by midnight of the previous day. Each dataset is recorded in the National Catalogue of Open Data, and is therefore fully trackable in the central catalogue system. The main benefits and added values of this catalogue include the possibility of full-text and parametric search, a direct interconnection with the National Catalogue of Open Data of the Czech Republic, and the necessary support for international standards in the publication of open data. Moreover, users can employ these open data to reconstruct almost all summaries published on the web interface *COVID-19*: *An Overview of the Current Situation in the Czech Republic*. The overview of the datasets including their structure and description is detailed in S1 Table.

- Epidemiology—datasets based on everyday reports on newly confirmed and terminated cases of the COVID-19 infection submitted to the ISID system by RPHAs and laboratories.
- Tests—daily and cumulative data on the numbers of performed antigen tests according to type, indication, and region/district.
- Vaccination—distribution and consumption of COVID-19 vaccines number of vaccinated people according to vaccination centres and profession.
- Hospital care availability mapping—daily changes in the available hospital capacity in the individual regions (the total number of available beds, machines, personnel).
- Distribution of personal protective equipment (PPE)—data about the distribution of PPE (glasses, disinfectants, masks, respirators, etc.) from the Administration of National Material Reserves to individual regions.

#### Restricted-access COVID-19 data

Datasets for predictive modelling—secured datasets covering the period from the beginning of the epidemic to the date one week before the current date. The one-week delay allows publication of a comprehensive, fully validated dataset; the delay is intentional because after the original report on positivity, supplementary data can be added to the original records, or the records can be amended to make them more accurate (e.g. the patient’s place of residence) (S2 Table).

Datasets for regions of the Czech Republic: Administration of municipalities with extended competencies (MEC)—datasets intended for the MEC administrations includes a complex overview of the basic epidemiological parameters with a special focus on the vulnerable elderly population groups (65+, 75+) at the geographical level of the individual MECs and districts; selected data are also available at the level of individual municipalities (S3 Table).

Datasets for crisis management of the Czech Republic at the national level—a set of daily reports documenting the current condition and course of the COVID-19 epidemic in the Czech Republic to regularly supply the top management of the Czech healthcare system and of the Integrated Central Crisis Management Team of the Ministry of Health with the latest data, including the current capacities of acute hospital-based care from the Intensive Care Control Centre [20] (S3 Table).

### Reports, summaries, and online interactive visualisations of available data

This section provides an overview of selected outputs of individual datasets and their real usage in practice at both regional and national levels. These include web applications primarily intended for the general and expert public, media, and news channels. Besides the MH, representatives of cities, regions, news servers, and other subjects participated in the development of these systems.

#### COVID-19: The overview of the current situation in the Czech Republic

**Fig 3:**
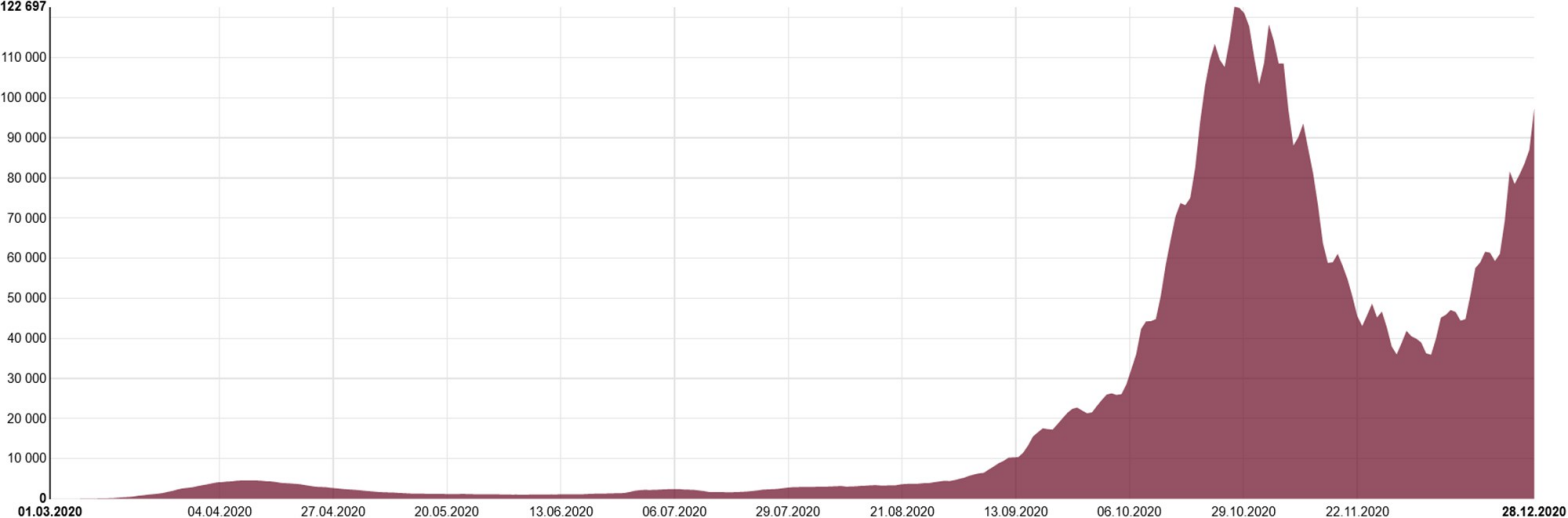
The trend profile of the COVID-19 active cases in the Czech Republic.

An official Czech website guaranteed by the Ministry of Health provides the statistics and interactive presentation of the COVID-19 epidemic in the country, as well as time series and cumulative overviews from its beginning (see Fig 3) [7]. Data are updated on a daily basis from the Information System of Infectious Diseases. The tool has been widely used by media, national and regional authorities, and the general public [15].

#### Dashboard for Integrated Central Crisis Management Team of the Ministry of Health

The Dashboard [21] is a management tool for the Integrated Central Management Team providing a real-time analytical overview and data sharing. This tool, along with the data sources, provides complex analytical reporting for the government of the Czech Republic and the supporting workgroups and predictive models for mathematical determination of various scenarios of the epidemic. The application provides the user with the option to simply change the graph parameters and to show data based on visual filters (regions, districts, districts of Prague, age groups, etc.). Access to this application is restricted to the members of the government, top management of MH and of workgroups participating in the crisis management, representatives of the Czech Armed Forces, and other involved institutions.

#### Predictive models

**Fig 4:**
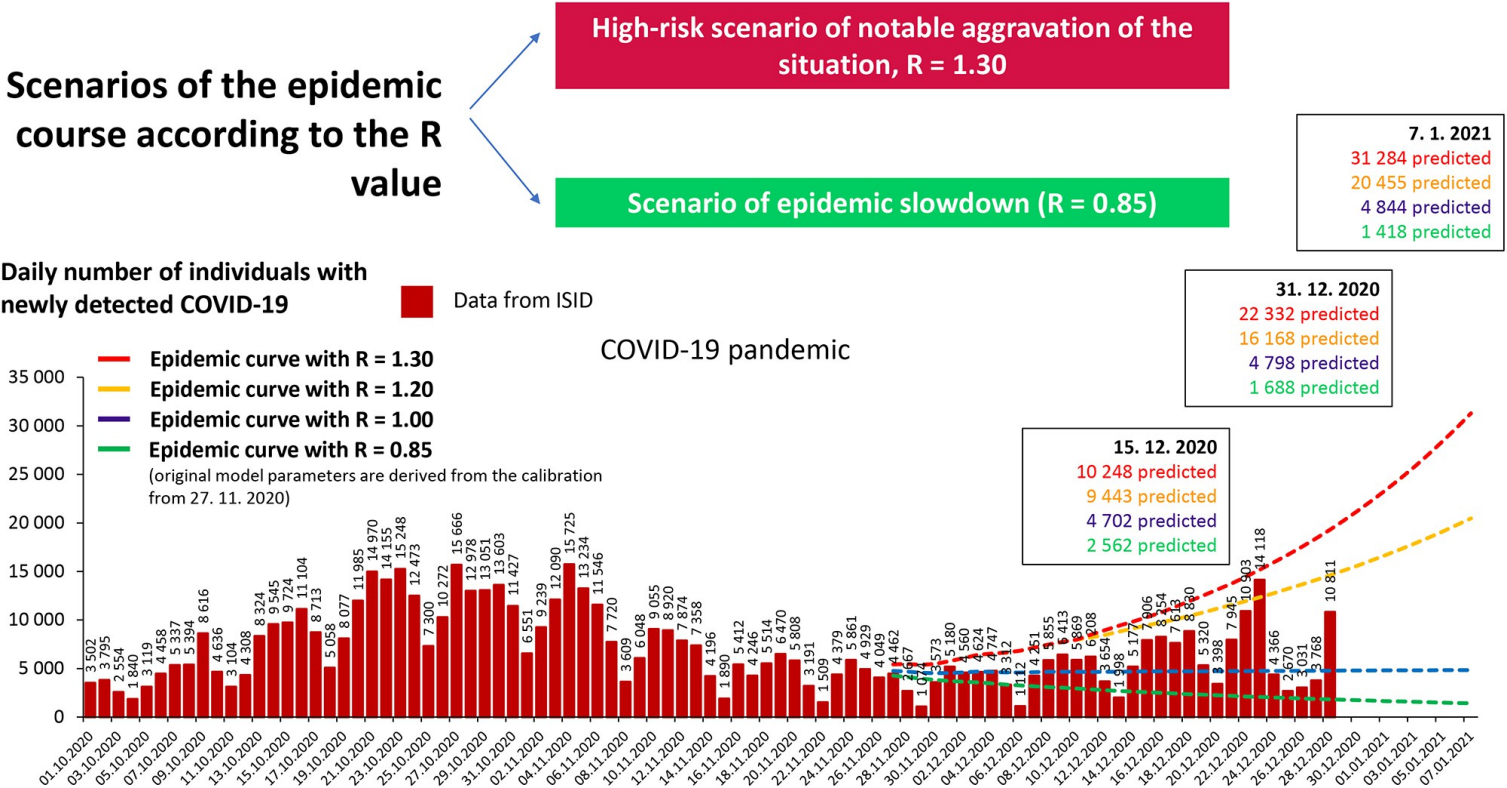
Situation in the entire Czech population: Possible scenarios for various values of the reproduction number R.

A simple epidemiological model [22] was based on published dataset describing current epidemiological situation in the Czech Republic and developed at the Institute of Health Information and Statistics of the Czech Republic to help decision-makers understand the course of the epidemics (including the estimation of the effective reproduction number) and to facilitate short-term predictions. The model uses the classical S(E)IR approach [23] with the following compartments: S (susceptible), I (infected, set of compartments), R_subcl_ (subclinical cases), and R (removed, laboratory-confirmed COVID-19 cases). In addition, a more complex SEIR model was adapted [24] and is being used for the explanation and forecasting of the COVID-19 epidemic (see Fig 4).

#### The use of open data by other bodies

Nowadays, many third-party services are connected directly on selected open data, using a permanent URL address of an API secure token. All these overviews and summaries are built on the datasets introduced in the section Public datasets. The Golemio Data Platform [25] is a set of technical tools for integration, storage, visualisation, and sharing of data managed by experts on municipal data. The Golemio Data Platform, which is built on the Microsoft Power BI technology, aims to provide quality IT services to the City and District councils in the field of Smart City data processing. Novinky.cz [26], one of the leading news servers in the Czech Republic, uses the Flourish platform for visualisation of the coronavirus spreading in the individual municipalities and Prague districts. The same technology is used e.g. by the BBC, Bosch company, or British Council. Another example of the work with open data is the authenticated map visualisation operated by the Association of Local Councils of the Czech Republic (website is accessible only for ArcGisMembers). Through map layers and the ESRI (Environmental Systems Research Institute) technology, this visualisation clearly depicts the current situation in the municipalities of the Czech Republic and the social services threatened by the COVID-19 disease. The overview allows the visualisation of nursing homes and retirement homes in relation to the current number of COVID-19 cases in the respective municipality.

### Statistical overview of the access to the datasets

The datasets are available through an application interface (API) [19] on the platform *COVID-19*: *An Overview of the Current Situation in the Czech Republic*. It is one of the access points to the datasets provided in the context of the COVID-19 epidemic. The API represents an alternative access point to the datasets published in the National Catalogue of Open Data of the Czech Republic and is optimised for automated data processing. Recently implemented functions include, e.g., the introduction of improved data compression, enabling the use of the resources by other domains (Cross-Origin Resource Sharing), minification of the datasets, unification, and standardisation of the attribute names, etc.

Most datasets are provided in the CSV and JSON data formats (including the minified version). The open data schema is described in accordance with the best practices of the standardised open data publication.

As of 30 June 2021, a total of 673 registered users were permitted to use the restricted-access datasets. There were 261 representatives of national, regional and local authorities and crisis management teams, and 412 users accessing data for predictive modelling. Researchers from Czech and foreign universities were the most frequent type of users working with data modelling (34%), followed by private persons (31%), private companies (17%), public sector and institutions (10%) and media (8%). The total number of API calls from 30 September 2020 to 30 June 2021 was 861,705; the *hospitalised patients* being the most popular dataset with 323,004 calls. A total of 56 datasets have already been published on the COVID-19 epidemic, describing comprehensively the current situation in the Czech Republic (the smallest dataset “Basic overview” has 4 kB, the largest dataset “Vaccination according to profession” has 3.2 GB). Additional datasets are constantly being prepared in response to new stakeholders’ and users’ requirements and requests.

## Discussion

The presented framework covering open data as well as secured datasets has been widely used by the general public, researchers, public authorities and decision makers. These datasets have been adopted as an official and guaranteed source for outputs provided by third parties, including public authorities, non-governmental organisations, researchers and online news portals.

Existing platforms and information systems can be employed to enhance emergency risk communication. Information and communication systems should be tailored to users’ needs and involve local stakeholders to guarantee the flow of information across sectors [27]. From our perspective, there are several major points in which official and validated national datasets on COVID-19 epidemic have played a crucial role. They support communication from national, regional, and local authorities to the general public, explaining the current situation and adopted measures. Since the datasets have been used by different types of users (from individual data enthusiasts over companies and institutions to the government and mainstream media), the same information reaches different target populations through various communication channels. One of the secondary aims of the authors has therefore been setting up a community interested in processing, analysis and visualisation of health data in order to maximise the impact of these activities across stakeholders, media, and the general public. Based on our long-term experience with involved users and stakeholders in the field of health data, we have identified the following major technical and methodological limitations that complicated the proper and smooth use of published data: (i) The size of selected data files in combination with MS Excel tool that do not allow their retrieval and subsequent processing. (ii) A metadata description in a machine-readable and standardised format that several users have not been able to find and understand. (iii) Reverse changes in data related to data collection and reporting methodology caused distrust and misunderstanding of published information. (iv) The inability to correctly interpret data by representatives of selected media channels due to their lack of understanding of data collection methodology and the importance of epidemiological characteristics.

An extension of existing datasets and the publication of new ones have closely followed the development of the COVID-19 epidemic, the requirements of national and local authorities and the interest of the general public—starting with overviews of the epidemic in spring 2020, over numbers of hospitalisations and availability of intensive care during the autumn 2020 surge, to the progress in vaccination in early 2021. There has been a high demand by media and data analysts for open datasets on a particular agenda right after its start. The preparation of a properly structured and, above all, validated dataset always requires an individual assessment. It must be thoroughly assessed what information the dataset contains, including the level of detail (with respect to the EU General Data Protection Regulation). With any combination of filters over the individual attributes of the dataset, the number of records found must be at least 10, otherwise a risk might occur that a specific person would be traceable. The mere existence of source data is far from being equivalent to publishing a dataset. The complete listing of source databases should always be carefully checked, bearing in mind the subsequent automation in export, preprocessing, analytic procedures, and the deployment of final publication scripts. If the above prerequisites are met, the dataset can be fully published as open data. Some data are included in internal decision-making systems and their publication may be subject to different processes and approvals. This is often in contradiction with requirements for an “absolute” openness and building trust of professionals and the general public. In particular, this was the case of data on the availability of acute care in individual hospitals, or data on the vaccination process in different types of healthcare providers. Thus, if a publication of any dataset is planned, its design and preparation should be an integral part of the whole agenda or process of collection and publication of new data.

Datasets currently published as open data meet the 3-star open data requirements, which makes them machine-readable and facilitates their further usage without restrictions. This is essential for making the data more easily understandable and usable for data consumers. Nevertheless, most datasets contain also geographical data, typically used for territorial differentiation. Such data need additional processing or context. Hence, transformation of these datasets to more complex RDF formats is planned as these formats support/facilitate contextualisation by linking to existing resources. The current course of the epidemics makes the importance of such datasets ever more and more apparent and hence, our goal is to proceed to publish 5-star linked open data in the future versions of the API. This approach will allow us to ensure the necessary interoperability and thus remove the limitations associated with dataset mapping and interconnection. We are moving towards a state where it will be possible to easily interconnect different systems at the data layer, which is absolutely crucial for an effective communication among various web applications.

Personal data processing and ethics are always an important issue in acquisition of health data based on particular method of publishing and sharing. Each dataset must be individually proposed, designed, analysed and validated. Moreover it must comply with general regulations (such as General Data Processing Regulation in EU countries) and national legislation, which are sometimes in contradiction with requirements for data openness and detailedness. This is the reason why three different methods of publishing and sharing have been defined. The mode of publication and legally regulated data availability is clearly defined for each dataset from the National Health Information System; in this regard, the law distinguishes among non-public data, data accessible only to legally defined readers/editors, reference statistical data for identified purposes and identified applicants, and statistical data published in the form of open datasets. For example, too detailed information about a person’s sex, age and place of residence can lead to his/her identification, particularly in small communities. It is therefore important to consider the proper degree of geographical (region, district, municipality) and demographic (specific age vs. age group) type of information that is published. Open data represent the publication of secondarily produced datasets and processed statistics, which must always comply with the legislation in force.

Based on literature review, documented and shared public health data can help to prevent catastrophic events. Such data can also help health researchers and, by extension, improve health status of a city, a state, a nation, or the world at large [28]. In the current COVID-19 pandemic, the need to share and collaborate openly has proved to be more important than personal careers or organisational goals [29]. The COVID-19 pandemic has made it clear that the health informatics community agrees with and strongly demands unified frameworks for sharing and exchanging digital epidemiological data and, accordingly with data protection regulations, facilitating the flow of information between health workers, stakeholders, policy makers and the public. The demand for digital data sharing has also raised some crucial points of discussion [30]. Nowadays, many country-specific open data guidelines have been created (e.g. in the United States [31] or Canada [32]). The publication of data in the Czech healthcare sector as an integral part of building e-government—and ensuring the necessary interoperability across the European Union—is based on methodological recommendations under the guarantee of the Department of the Chief Architect of eGovernment of the Ministry of the Interior of the Czech Republic. Specifically, this includes the strategic framework Czech Republic 2030, the action plan for Society 4.0 and the legal regulation of open data in the form of Act No. 106/1999 Coll., on free access to information [33]. In accordance with the strategic document EU implementation of the G8 Open Data Charter [34], essential datasets with maximum information added value for users at national and EU level are identified and subsequently published with the aim of prospective linking with the EU Open Data Portal. As part of a long-term effort to publish not only COVID-19 data, internationally valid recommendations such as the FAIR principles [35], which stress various preconditions for data sharing and emphasise the methodological and transparency requirements in reporting scientific research into the domain of data stewardship [36], are being considered.

Data sharing is one of the pillars of scientific progress, and cooperation between different countries and cultures is the fastest way to accumulate valuable knowledge and face challenges such as the current pandemic [37]. In conjunction with the strategy of the MH in the field of data opening, additional datasets meeting the already implemented standards will be also released, both on COVID-19 related and unrelated topics. The Ministry and its subsidiary organisations possess an immense amount of data in the National Health Information System, which contains long-term records in tens of registries. The plan is to gradually make data of these registries available over the next few years by automated publishing of selected open datasets that will go through the full life-cycle of the dataset according to the given methodology (see section Methods).

## Supporting information

S1 Table Content of public open datasets on COVID-19 in the Czech Republic. (DOCX)

S2 Table Content of datasets on COVID-19 in the Czech Republic for predictive modelling. (DOCX)

S3 Table Content of regional datasets on COVID-19 in the Czech Republic for local authorities and regional disease management. (DOCX)

## References

[1] Altayar MS. Motivations for open data adoption: An institutional theory perspective. Gov Inform Q. 2018 Oct;35(4):633–643. doi: 10.1016/j.giq.2018.09.006

[2] Our World in Data [Internet]. 2020 [Cited 2021 Jun 28]. Coronavirus pandemic (COVID-19)—the data. https://ourworldindata.org/coronavirus-data.

[3] European Centre for Disease Prevention and Control [Internet]. 2020 [Cited 2021 Jun 28]. COVID-19 Coronavirus Data—Weekly (from 17 December 2020. https://data.europa.eu/euodp/en/data/dataset/covid-19-coronavirus-data-weekly-from-17-december-2020.

[4] Dong E, Du H, Gardner L. An interactive web-based dashboard to track COVID-19 in real time. Lancet Infect Dis. 2020 May;20(5):533–534. doi: 10.1016/S1473-3099(20)30120-1 32087114 PMC7159018

[5] CSSEGISandData [Internet]. 2020 [Cited 2021 Jun 28]. COVID-19 Data Repository by the Center for Systems Science and Engineering (CSSE) at Johns Hopkins University. https://github.com/CSSEGISandData/COVID-19.

[6] COVID-19 Data Hub [Internet]. 2020 [Cited 2021 Jun 28]. https://github.com/covid19datahub/COVID19.

[7] Komenda M, Karolyi M, Bulhart V, Žofka J, Brauner T, Hak J, et al. COVID-19: Overview of Current Situation in the Czech Republic. Diseases at the Moment. Prague: Ministry of Health of the Czech Republic. 2020 [Cited 2020-12-30]. https://onemocneni-aktualne.mzcr.cz/covid-19.

[8] Centers for Disease Control and Prevention [Internet]. 2020 [Cited 2021 Jun 25]. CDC COVID Data Tracker. https://covid.cdc.gov/covid-data-tracker/.

[9] Etalab [Internet]. 2020 [Cited 2021 Jun 25]. Coronavirus epidemic monitoring dashboard in France. https://www.data.gouv.fr/en/reuses/tableau-de-bord-de-suivi-de-lepidemie-de-coronavirus-en-france/.

[10] Robert Koch Institute [Internet]. 2020 [Cited 2021 Jun 25]. COVID-19 (Coronavirus SARS-CoV-2). https://www.rki.de/DE/Content/InfAZ/N/Neuartiges_Coronavirus/nCoV.html.

[11] Federal Ministry for Digitization and Business Location [Internet]. Austrian COVID-19 Open Data Information Portal [Cited 2021 Jun 25]. https://www.data.gv.at/covid-19/.

[12] Open Data Initiative of the Government of Spain [Internet]. [Cited 2021 Jun 25]. https://datos.gob.es/.

[13] Public Health England [Internet]. Coronavirus (COVID-19) in the UK [Cited 2021 Jun 25]. https://coronavirus.data.gov.uk.

[14] Alamo T, Reina DG, Mammarella M, Abella A. Covid-19: Open-data resources for monitoring, modeling, and forecasting the epidemic. Electronics 2020;9(5):827. doi: 10.3390/electronics9050827

[15] Komenda M, Bulhart V, Karolyi M, Jarkovský J, Mužík M, Májek O, et al. Complex reporting of coronavirus disease (COVID-19) epidemic in the Czech Republic: use of interactive web-based application in practice. J Med Internet Res. 2020 May;22(5):e19367. doi: 10.2196/19367 , 32412422 PMC7254961

[16] Ministry of Health of the Czech Republic [Internet]. 2019 [Cited 2021 May 14]. Open Data Catalogue. https://opendata.mzcr.cz/.

[17] Ministry of Interior of the Czech Republic [Internet]. 2018 [Cited 2021 May 14]. National Catalogue of Open Data. https://data.gov.cz/datasets.

[18] Ministry of Health of the Czech Republic [Internet]. 2020 [Cited 2021 May 14]. Diseases at the Moment: Overview of Current Situation in the Czech Republic. https://onemocneni-aktualne.mzcr.cz/.

[19] Ministry of Health of the Czech Republic [Internet]. 2020 [Cited 2021 May 14]. COVID-19 in the Czech Republic: Open datasets and datasets for download. https://onemocneni-aktualne.mzcr.cz/api/v2/covid-19.

[20] Ministry of Health of the Czech Republic [Internet]. 2020 [Cited 2021 May 16]. COVID-19: Online Controlling of Intensive Care. https://dip.mzcr.cz/.

[21] Ministry of Health of the Czech Republic [Internet]. 2020 [Cited 2021 May 14]. COVID-19 Dashboard. https://dashboard.uzis.cz.

[22] Májek O, Ngo O, Jarkovský J, Komenda M, Rážová J, Dušek L, et al. 2020. Modelling the first wave of the COVID-19 epidemic in the Czech Republic and the role of government interventions. Available from: https://www.medrxiv.org/content/10.1101/2020.09.10.20192070v1.

[23] Kucharski AJ, Russell TW, Diamond C, Liu Y, Edmunds J, Funk S, et al. Early dynamics of transmission and control of COVID-19: a mathematical modelling study. Lancet Infect Dis. 2020 May;20(5):553–558. doi: 10.1016/S1473-3099(20)30144-4 32171059 PMC7158569

[24] Prem K, Liu Y, Russell TW, Kucharski AJ, Eggo RM, Davies N, et al. The effect of control strategies to reduce social mixing on outcomes of the COVID-19 epidemic in Wuhan, China: a modelling study. Lancet Public Health. 2020 May;5(5):e261–e270. doi: 10.1016/S2468-2667(20)30073-6 32220655 PMC7158905

[25] Golemio Data Platform [Internet]. [Cited 2021 Jun 19]. https://golemio.cz/en.

[26] Novinky cz [Internet]. [Cited 2021 Jun 19]. https://www.novinky.cz/

[27] World Health Organization. Communicating risk in public health emergencies: a WHO guideline for emergency risk communication (ERC) policy and practice. Geneva: World Health Organization; 2017. ISBN 978-92-4-155020-8. 31059213

[28] D’Agostino M, Samuel NO, Sarol MJ, de Cosio FG, Marti M, Luo T, et al. Open data and public health. Rev Panam Salud Publica. 2018; 42: e66. doi: 10.26633/RPSP.2018.66 31093094 PMC6386141

[29] Park JJH, Mogg R, Smith GE, Nakimuli-Mpungu E, Jehan F, Rayner CR, et al. How COVID-19 has fundamentally changed clinical research in global health. Lancet Glob Health. 2021;May;9(5):e711–e720. doi: 10.1016/S2214-109X(20)30542-8 33865476 PMC8049590

[30] Dagliati A, Malovini A, Tibollo V, Bellazzi R. Health informatics and EHR to support clinical research in the COVID-19 pandemic: an overview. Brief Bioinform. 2021 Mar 22;22(2):812–822. doi: 10.1093/bib/bbaa418 33454728 PMC7929411

[31] Government of the USA [Internet]. [Cited 2021 Jun 19]. The home of the U.S. Government’s open data. https://www.data.gov/.

[32] Government of Canada [Internet]. [Cited 2021 Jun 19]. Open Data. https://open.canada.ca/en/open-data.

[33] Legislativní prostředí otevřených dat [Legislative environment of open data] [Internet]. [Cited 2021 Jun 19]. https://opendata.gov.cz/legislativa:start.

[34] European Commission [Internet]. [Cited 2021 Jun 24]. EU implementation of the G8 Open Data Charter. https://digital-strategy.ec.europa.eu/en/library/eu-implementation-g8-open-data-charter.

[35] Wilkinson MD, Dumontier M, Aalbersberg IJJ, Appleton G, Axton M, Baak A, et al. The FAIR Guiding Principles for scientific data management and stewardship. Sci Data. 2016 Mar 15;3:160018. doi: 10.1038/sdata.2016.18 26978244 PMC4792175

[36] Boeckhout M, Zielhuis GA, Bredenoord AL. The FAIR guiding principles for data stewardship: fair enough? Eur J Hum Genet. 2018 Jul;26(7):931–936. doi: 10.1038/s41431-018-0160-0 29777206 PMC6018669

[37] Rios RS, Zheng KI, Zheng MH. Data sharing during COVID-19 pandemic: what to take away. Expert Rev Gastroenterol Hepatol. 2020 Dec;14(12):1125–1130. doi: 10.1080/17474124.2020.1815533 32842793

