# Supplementary material for: Sharing datasets of the COVID-19 epidemic in the Czech Republic
Source: PLoS One. 2022 Apr 21;17(4):e0267397. doi: 10.1371/journal.pone.0267397 (PMC9022808; doi:10.1371/journal.pone.0267397)
Supplement: S1 Table — (DOCX) [file pone.0267397.s001.docx]

**Table 1. Content of public open datasets on COVID-19 in the Czech Republic**

| **Dataset** | **Description** | **Content** |
| --- | --- | --- |
| **Epidemiology** | | |
| Basic overview | Basic characteristics and parameters published on a daily basis | Date  Total and newly performed tests  Total and new confirmed cases  Total and new confirmed cases 65+  Active cases  Recovered  Deaths  Currently hospitalised  Performed antigen tests  Total and new vaccinations |
| Overview of individuals with proved infection according to the RPHA reports | Basic daily overview of individuals who were proved to suffer from COVID-19 according to the RPHA reports (report date, age, and sex of the infected person, the reporting RPHA, information about the place and country of contagion). | Date  Age  Sex  Reporting RPHA  Place of contagion  Country of contagion |
| Overview of recovered patients according to the RPHA reports | Records about patients recovered from the COVID-19 disease according to the RPHA reports. As the recovery data are somewhat delayed due to the necessary validation and case closing at the RPHAs, the daily records may be subject to retrospective amendments caused by this verification. | Date  Age  Sex  Region (NUTS 4)  District (LAU) |
| Overview of deaths according to the RPHA reports | Records about deaths of COVID-19 patients according to the RPHA reports. It contains information about deaths of individuals positively tested for SARS-CoV-2 (by PCR) regardless of the reasons for their deaths, and regardless of whether they died in a hospital or outside hospital care. As the mortality data are somewhat delayed due to the necessary validation and case closing at the RPHAs, the daily records may be subject to retrospective amendments caused by this verification. | Date  Age  Sex  Region (NUTS 4)  District (LAU) |
| Overview of hospitalisations | Description of the hospitalisation course of COVID-19 patients (current and total number of hospitalised patients, discrimination according to symptoms severity and medical equipment used, number of deaths) | Date  Total, ongoing, and new hospitalisations  Disease severity  Medical equipment  Deaths and cumulative deaths |
| Total (cumulative) number of individuals with proven infection according to reports of the RPHAs and laboratories, number of recovered patients, deceased patients, and tests | Daily cumulative numbers of individuals with proven COVID-19 infection according to reports of the RPHAs and laboratories, number of recovered patients according to the RPHA reports, numbers of deceased SARS-CoV-2 positive patients, and daily numbers of the performed tests on COVID-19 according to the lab reports. As the mortality data are somewhat delayed due to the necessary validation and case closing at the RPHAs, the daily records may be subject to retrospective amendments caused by this verification. | Date  Cumulative and incremental number of infected individuals  Cumulative and incremental number of recovered individuals  Cumulative and incremental number of deaths  Cumulative and incremental number of tests performed  Cumulative and incremental number of antigen tests performed |
| Overview of the epidemiological situation according to the RPHA reports and district | Cumulative and daily numbers of individuals with proved COVID-19 infection according to the validated RPHA reports, cumulative recovered COVID-19 patients according to the RPHA reports, cumulative daily numbers of deaths of patients with COVID-19 according to the RPHA reports, and of hospital deaths, stratified by districts and regions. As the mortality and recovery data are somewhat delayed due to the necessary validation and case closing at the RPHAs, the daily records may be subject to retrospective amendments caused by this verification. To allow proper validation by the RPHAs and to minimise these retrospective amendments, the dataset is always published with a 1-week delay. | Date  Region (NUTS 4)  District (LAU)  Cumulative number of infected individuals  Cumulative number of recovered individuals  Cumulative number of deaths |
| Overview of the epidemiological situation according to the RPHA reports and municipalities with extended competencies (MEC) | Complex overview of basic epidemiological parameters (new confirmed cases, active cases, hospitalised patients) with a focus on vulnerable groups (age over 65 and 75 years) stratified by MECs | Date  MEC  New cases in last 7 days  New cases in last 7 days in 65+  New cases in last 7 days in 75+  Active cases  Active cases in 65+  Active cases in 75+  Number of hospitalised patients  Number of new hospitalisations in last 7 days  Number of tests performed in last 7 days |
| Epidemiologic characteristics of municipalities | Complex overview of basic epidemiological parameters stratified by MECs | Date  Municipality  New confirmed cases  Active cases  New cases in 65+  New cases in last 7 days  New cases in last 14 days |
| Epidemiologic characteristics of Prague and its districts | Complex overview of basic epidemiological parameters stratified by districts of Prague | Date  Municipality  New confirmed cases  Active cases  New cases in 65+  New cases in last 7 days  New cases in last 14 days |
| Total number of individuals with proven infection according to reports of the RPHAs and laboratories in last 7 and 14 days in the Czech Republic | Number of confirmed cases in last 7 and 14 days in the entire country, including standardisation per 100,000 persons. An open dataset of the Czech Statistical Office *Population according to age and sex in regions and districts* is used for the standardisation. | Date  New cases in last 7 days  New cases in last 14 days  New cases in last 7 days per 100,000 persons  New cases in last 14 days per 100,000 persons |
| Total number of individuals with proven infection according to reports of the RPHAs and laboratories in last 7 and 14 days in regions | Number of confirmed cases in last 7 and 14 days in Czech regions, including standardisation per 100,000 persons. An open dataset of the Czech Statistical Office *Population according to age and sex in regions and districts* is used for the standardisation. | Date  New cases in last 7 days  New cases in last 14 days  New cases in last 7 days per 100,000 persons  New cases in last 14 days per 100,000 persons |
| Total number of individuals with proven infection according to reports of the RPHAs and laboratories in last 7 and 14 days in counties | Number of confirmed cases in last 7 and 14 days in Czech counties, including standardisation per 100,000 persons. An open dataset of the Czech Statistical Office *Population according to age and sex in regions and districts* is used for the standardisation. | Date  New cases in last 7 days  New cases in last 14 days  New cases in last 7 days per 100,000 persons  New cases in last 14 days per 100,000 persons |
| **Tests** | | |
| Overview of performed tests according to type and indication | Daily numbers of tests stratified with the discrimination of RT-PCR tests and antigen tests as reported by laboratories. The dataset further contains numbers of tests according to indication (diagnostic, epidemiological, preventive, other) and number of positive cases according to type and indication | Date  Number of RT-PCR and antigen tests  Number of tests according to indication  Number of positive tests  Number of positive tests according to indication  Number of positive tests according to type |
| Total (cumulative) numbers of performed tests in regions and districts | Incremental and cumulative numbers of RT-PCR tests corrected for repeated positive tests as reported by laboratories, without regard to the type of reimbursement. Antigen tests are not included. The dataset is available in detailed structure from 1 August 2020 and in aggregated form by 31 July 2020. | Date  Region, district  Incremental number of performed tests in a region/district  Cumulative number of performed tests in a region/district  Incremental number of first tests in a region/district  Cumulative number of first tests in a region/district |
| Testing facilities | A list of public testing points that perform RT-PCR and antigen testing for SARS-CoV-2. Facilities that perform tests e.g. before patient’s admission to hospital-based care are not included. | Facility name  District  Address  Coordinates  Testing capacity  Nasopharyngeal sampling  Oropharyngeal sampling  Antigen testing  Drive-in |
| **Vaccination** | | |
| Vaccination in regions | Aggregated data about reported vaccinations in Czech regions. The data is stratified according to age and type of vaccine. | Date  Vaccine type  Region  Age group  Number of first, second, and total doses |
| Vaccination in vaccination centres | Aggregated data about reported vaccinations in individual vaccination centres. The data is stratified according to age and type of vaccine. | Date  Vaccine type  Region  Facility  First/second dose  Age group |
| List of vaccination centres | A list of public vaccination centres in the Czech Republic that provide vaccination against COVID-19. | Facility  District  Address  Coordinates  Type  Code  Minimum capacity  Wheelchair accessible |
| Consumption of vaccines in centres | Overview of consumption of individual types of vaccines (used and depreciated ampoules) | Date  Facility  Region  Type of vaccine  Manufacturer  Used ampoules  Applied doses  Depreciated doses |
| Distribution of vaccines | Overview of distribution of vaccines in individual vaccination centres – number of ampoules received and used for each type of vaccine | Date  Facility  Region  Target facility  Target region  Type of vaccine  Manufacturer  Action  Number of ampoules  Number of doses |
| Registrations for vaccination in centres | Overview of registrations in the central reservation system for COVID-19 vaccination. | Date  Facility  Region  Age group  Profession  Country  Reservation  Date of reservation  State of registration  Priority group  Blocking and reason of blocking |
| Reservations for vaccination in centres | Free and maximum capacities in vaccination centres in individual days reported from the central reservation system. | Date  Facility  Region  Free capacity  Maximum capacity  Vaccination calendar |
| Vaccination according to profession | Reported vaccinations according to profession in individual vaccination centres. | Date  Type of vaccine  Region  Facility  First/second dose  Profession / priority group |
| Vaccination points | List of vaccination points supplementary to the list of vaccination centres – general practitioners and others. | Facility  Region  District  Establisher  General practitioner |
|  |  |  |
|  |  |  |
| **Mapping of the availability/capacity of intensive care** | | |
| Online Control Centre for Intensive Care – available capacities | A dataset stratified by regions of the Czech Republic, containing daily overviews of the changes in the available capacities of the acute hospital care (total numbers of the available machines, beds, and personnel, together with available capacity on individual days) available for COVID-19 patients. The dataset contains data about the available machine equipment (ECMO, ventilators, CRRT, IHD), bed occupancy (only adult ICU+HDU beds and oxygen-equipped standard beds per hospital), and about the available personnel (only doctors and nurses on adult ICUs and HDUs). | Date  Region  Machine equipment / type of beds  Free capacity – COVID-negative  Occupied capacity – COVID- negative  Free capacity – COVID-positive  Occupied capacity – COVID-positive |
| Online Control Centre for Intensive Care – available capacities in individual healthcare facilities. | Daily changes of available capacities in hospitals that provide acute care. Two datasets before and from 1 April 2021 are available. | Date  Facility  Region  Machine equipment / type of beds  Free capacity – COVID-negative  Occupied capacity – COVID- negative  Free capacity – COVID-positive  Occupied capacity – COVID-positive |
| **Distribution of protective equipment** | | |
| The overview of the distribution of protective equipment by regions of the Czech Republic | A dataset containing the up-to-date overview of the numbers of protective equipment distributed into the regions of the Czech Republic on individual days (glasses, disinfectants, masks, respirators, etc.) | Date  Type of equipment  Region  Number of items |
