# Supplementary material for: Sharing datasets of the COVID-19 epidemic in the Czech Republic
Source: PLoS One. 2022 Apr 21;17(4):e0267397. doi: 10.1371/journal.pone.0267397 (PMC9022808; doi:10.1371/journal.pone.0267397)
Supplement: S2 Table — (DOCX) [file pone.0267397.s002.docx]

**Table 2. Content of datasets on COVID-19 in the Czech Republic for predictive modelling**

| **Dataset** | **Description** | **Content** |
| --- | --- | --- |
| Infected, recovered, and deceased patients at the district level | This dataset contains aggregated data about the number of SARS-Cov-2 positive patients, recovered patients, and SARS-Cov-2 positive deceased patients on individual days, stratified by district of residence, sex, and age category based on the reports by RPHAs, laboratories, and hospitals. | Date (of the positive test, recovery, or death)  District in the CZ-NUTS format  Sex  Age category (0-19, 20-64, and 65+ years)  Number of positive patients  Number of recovered patients  Number of deceased patients  Number of patients deceased in a hospital  Number of deceased with records of serious condition |
| Time effectiveness of testing | A dataset at the level of individual anonymised records of COVID-19 patients already processed by RPHA, containing time data about the periods between the onset of the symptoms, testing, test result, RPHA report, and isolating the positive patient as reported by the RPHAs and laboratories. | Date of first symptoms  Test date  Date of the result of the first positive PCR test  Date of RPHA report  Date of isolating the person |
| Re-testing of positive patients | The dataset contains aggregated data for individual days about the numbers of first positive tests, all positive tests, and positive re-tests based on lab reports. | Date  Number of first positive tests  Number of all positive tests  Number of re-tests in positive patients |
| Hospitalised patients | The dataset provides aggregated data about the numbers of hospitalised patients, aggregated by the district of residence, sex, age category, serious condition, and resulting patient condition according to hospital data supplemented with RPHA data. | District in the CZ-NUTS format  Sex  Age category (0-19, 20-64, and 65+ years)  Serious condition during hospitalisation  Resulting patient’s condition by the export date: released from the hospital, hospitalised, deceased  Number of patients |
| Time periods | Aggregated data about hospitalised patients aggregated on district, sex, age group, disease severity and recovery/death as reported by hospitals and supplemented by data from RPHAs. | District in the CZ-NUTS format  Municipalities with extended competence  Sex  Age category (0-19, 20-64, and 65+ years)  Date of first symptoms  Test date  Date of the result of the first positive PCR test  Date of RPHA report  Date of isolating the person  Date of admission to a hospital  Date of release from the hospital  Date of recovery  Date of death |
