# Supplementary material for: Sharing datasets of the COVID-19 epidemic in the Czech Republic
Source: PLoS One. 2022 Apr 21;17(4):e0267397. doi: 10.1371/journal.pone.0267397 (PMC9022808; doi:10.1371/journal.pone.0267397)
Supplement: S3 Table — (DOCX) [file pone.0267397.s003.docx]

**Table 3. Content of regional datasets on COVID-19 in the Czech Republic for local authorities and regional disease management**

| **Dataset** | **Description** | **Content** |
| --- | --- | --- |
| Weekly epidemiological characteristics for the authorities of municipalities with extended competence | Epidemiological characteristics (numbers of new and active cases, hospitalisations) on the level of municipalities with a focus on risk groups (age 65+ and 75+ years | The respective week  Municipalities with extended competence  Newly diagnosed patients per week  Newly diagnosed patients aged 65+ per week  Newly diagnosed patients aged 75+ per week  Newly diagnosed patients per week (according to the place of residence in individual ORPs  Current (on the last day of the particular week) number of patients with COVID-19  Current (on the last day of the particular week) number of patients aged 65+ with COVID-19  Current (on the last day of the particular week) number of patients aged 75+ with COVID-19  Current (on the last day of the particular week) number of patients hospitalised with COVID-19  (stratified by place of residence in a given ORP)  Number of performed PCR tests (stratified by the place of residence in a given ORP) |
| Weekly epidemiological characteristics of districts | Epidemiological characteristics (numbers of new and active cases, hospitalisations) on the level of districts with a focus on risk groups (age 65+ and 75+ years | The respective week  District code  Newly diagnosed patients per week  Newly diagnosed patients aged 65+ per week  Newly diagnosed patients aged 75+ per week  Newly hospitalised patients per week (stratified by district of residence)  Current (on the last day of the particular week) number of patients with COVID-19  Current (on the last day of the particular week) number of patients aged 65+ with COVID-19  Current (on the last day of the particular week) number of patients aged 75+ with COVID-19  Current (on the last day of the particular week) number of patients hospitalised with COVID-19 (stratified by district of residence)  Number of performed tests (stratified by the district of residence) |
| Basic epidemiological characteristics of municipalities | Basic epidemiological characteristics (new and active cases) on the level of municipalities | The respective week  District code  Municipality with extended competence  Municipality  Newly diagnosed per week  Current (on the last day of the particular week) number of patients with COVID-19 |
